# The Korean Baekdudaegan Mountains: A Glacial Refugium and a Biodiversity Hotspot That Needs to Be Conserved

**DOI:** 10.3389/fgene.2018.00489

**Published:** 2018-10-23

**Authors:** Mi Yoon Chung, Sungwon Son, Gang Uk Suh, Sonia Herrando-Moraira, Cheul Ho Lee, Jordi López-Pujol, Myong Gi Chung

**Affiliations:** ^1^Research Institute of Natural Science, Gyeongsang National University, Jinju, South Korea; ^2^Plant Conservation Division, Korea National Arboretum, Pocheon, South Korea; ^3^Botanic Institute of Barcelona (IBB, CSIC-ICUB), Barcelona, Spain; ^4^Division of Life Science and the Research Institute of Natural Science, Gyeongsang National University, Jinju, South Korea

**Keywords:** Baekdudaegan, conservation, Korean Peninsula, North Korea, South Korea

The Baekdudaegan (BDDG; Figure 1) is a mountain range relatively unknown outside Korea. From recent times, however, the BDDG is known outside Korea because it shelters the small county of Pyeongchang, the venue of the 2018 Winter Olympic Games. Within the Korean Peninsula, it is regarded as a sort of “backbone,” not only because it stretches across the whole peninsula with over 1,600 km (it is one of the longest chains of East Asia) but also because it is deeply embedded within the Koreans' spirituality (Choi, 2004; Mason, 2011; see also below). The BDDG is also well-known as a biodiversity hotspot, as it remains relatively pristine, particularly in South Korea. It harbors a very significant part of Korea's biota, especially regarding plants. It is estimated that just the South Korean part of this mountain range might include 1,500 plant species, i.e., about one third of the total flora of the Korean Peninsula (4,662 vascular plant species; Kim, 2006). About one hundred of the plant species native to the BDDG are endemic to the Korean Peninsula (Choi, 2004), with some of them being exclusively distributed within these mountains (e.g., *Gymnospermium microrrhychum, Hanabusaya asiatica, Megaleranthis saniculifolia*, and *Smilacina bicolor*; Figure 1). It should be also noted that the six genera generally regarded as endemic to Korea (*Abeliophyllum, Coreanomecon, Echinosophora, Hanabusaya, Megaleranthis*, and *Pentactina*; Kim, 2006; Kim et al., 2009) occur totally or partially within the BDDG and its vicinity. In addition, the new monotypic umbelliferous genus *Sillaphyton*, described in 2016, also occurs on a few calcareous localities on the central BDDG (Pimenov et al., 2016). Although animal surveys of the BDDG are not as complete as for plants, they suggest that this mountain range would be also very rich in species diversity. For example, the BDDG might boast up to 135 bird species (i.e., 26% of the total species for Korea), 36 mammal species (29%), and 32 species of amphibians and reptiles (60%) (Shin et al., 2016; percentages are calculated based on the total numbers for Korea of Lee and Miller-Rushing, 2014). As an another example, in only two national parks of the BDDG (Jirisan and Seoraksan) there are nearly 30% of the freshwater fish species of the Korean Peninsula (Jang et al., 2003). The BDDG is also a hotspot for threatened species; whereas most of the protected plant species of Korea have their populations within the BDDG mountain range (Kang et al., 2010), there are at least 30 endangered animal species that are included in the Convention on International Trade in Endangered Species (CITES; Cho and Chun, 2015).

**Figure 1:**
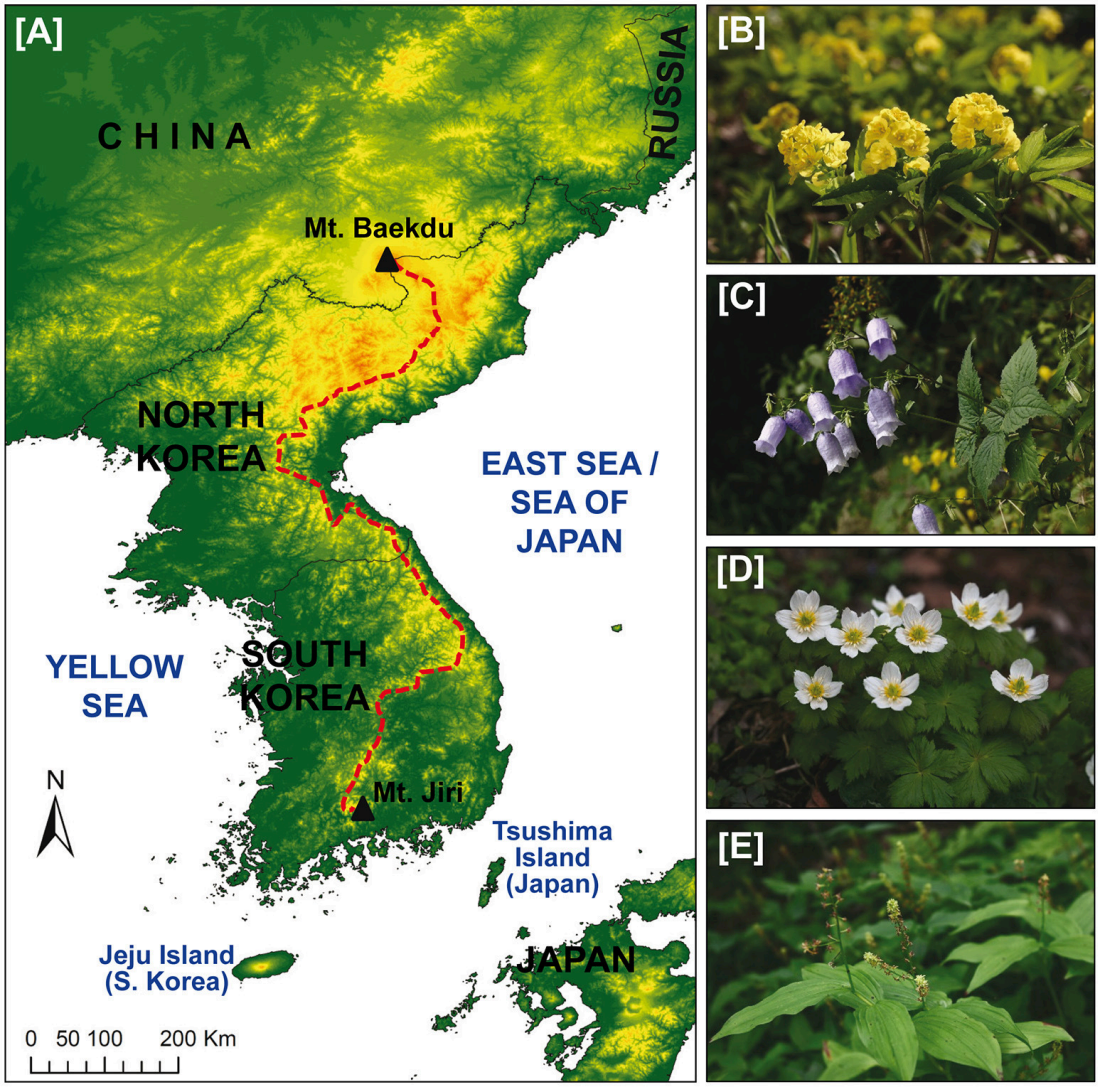
The BDDG mountain system and its representative plant species exclusively distributed. **(A)** Main ridgeline of the BDDG is represented by a red thick dashed line, **(B)** *Gymnospermium microrrhychum*, **(C)** *Hanabusaya asiatica*, **(D)** *Megaleranthis saniculifolia*, **(E)** *Smilacina bicolor*. The four photos were provided by Hyung Ho Yang (Seoul Botanic Park).

Such value as a biodiversity reservoir seems to be directly related to the role of the BDDG as a Pleistocene refugium, as shown by recent phylogeographic and palaeoecological studies (plant species reviewed in Chung et al., 2017; for animals see Borzée et al., 2017; Lee et al., 2018). Chung et al. (2017) critically reviewed the literature on genetic diversity and phylogeography of plants for which Korean populations were studied and found clear signals of the role of these mountains as a glacial refugium: [1] Korean populations showed higher intrapopulation genetic diversity than populations located further north (and, in some cases, with latitudinal decreases of genetic variation; i.e., consistent with the “southern richness” vs. “northern purity” paradigm of Quaternary biogeography; Hewitt, 2000; Hu et al., 2009); [2] Korean populations harbored ancestral haplotypes; and [3] Korean populations exhibited significant amounts of unique haplotypes/alleles (Chung et al., 2017). In addition, plant species whose studied range in Korea was mostly centered in the BDDG shared a clear pattern of high intrapopulation (%*P* = 46.0; *A* = 1.72; *H*_e_ = 0.159 with allozymes) and low to moderate interpopulation genetic variability (*G*_ST_ = 0.175, also with allozymes; Chung et al., 2017). The same authors also reviewed the available palaeoecological literature (mainly fossil pollen records and palaeovegetation reconstructions) which suggested that the BDDG sustained an assemblage of boreal and temperate forests at the Last Glacial Maximum (LGM), thus broadly supporting the genetic studies (Chung et al., 2017). A recently published study (Chung et al., 2018), focused on *Lilium cernuum*, also agrees with the BDDG refugium hypothesis, as populations sampled from these mountains harbored significantly higher genetic diversity than those located further north (in NE China); in addition, past distribution models (obtained with the maximum entropy algorithm implemented in MaxEnt; Phillips et al., 2006) showed higher probability of occurrence in southern ranges than in northern ones during the LGM. Similar results supporting the BDDG refugium hypothesis can be also found in endemic animals in East Asia. Kim et al. (2013) detected eight mitochondrial cytochrome *b* haplotypes from Korean populations (including some from the BDDG) of the raccoon dog *Nyctereutes procyonoides*, but only five from the Russian Far East. Borzée et al. (2017) found more mitochondrial haplotypes from populations of the Asian common toad (*Bufo gargarizans*) in the BDDG and its vicinity compared to other lowland localities (mean number of haplotypes; 3.33 vs. 2.43). Similarly, Jo et al. (2017), using five microsatellite loci, found that populations of the striped field mouse *(Apodemus agrarius)* on the BDDG and its vicinity harbor significantly higher levels of within-population genetic diversity than those from lower habitats (by excluding a population on Jeju Island; *H*_e_ = 0.864 vs. 0.805, *P* = 0.017, Mann-Whitney *U* test). Based on the mitochondrial cytochrome *b* gene, Lee et al. (2018) also suggested that populations in the southernmost part the Korean Peninsula, including the BDDG, played an important role as a refugium for the Asian lesser white-toothed shrew *(Crocidura shantungensis)* during the Pleistocene. The BDDG should be, thus, added to the list of the well-known East Asian Pleistocene refugia for plants and animals (e.g., the Hengduan Mts., the Nanling Mts., or the central China Mountains). On the basis of its shared role as a glacial refugium and a series of striking similarities in floristic richness and orographic features (length, orientation, altitude, and latitude), we believe that the BDDG would constitute a sort of “North East Asian counterpart” of the Southern Appalachians (Chung et al., 2016). Given its floristic, faunistic, and biogeographic value, therefore, the BDDG merits a high priority for conservation.

The present conservation status of these mountains show considerable differences, however, between the South and North Korean sections (reviewed in Chung et al., 2017). The southern part enjoys, in general, a good protection degree, as large parts are covered by the network of protected areas (PAs), including eight national and one provincial parks, and two Ramsar sites (Odaesan National Park Wetlands and The High Moor, Yongneup of Mt. Daeam). The setting up of the BDDG Mountains Reserve (BMR) in 2005 integrated these areas, in addition to adding 360 km^2^ of newly protected land, and it is expected to be extended by another *ca*. 700 km^2^ by the year 2020 (MOE, 2014). In 2018, the National Baekdudaegan Arboretum near the Sobaeksan National Park was opened to strengthen the BMR as a biological corridor and to harbor a seed vault for up to 2 million accessions. The Korea Forest Service is continuing efforts to set up new PAs and upgrade the status of already extant PAs (e.g., as UNESCO Biosphere Reserves). The northern part of the BDDG, in contrast, is rarely covered by PAs whereas deforestation might affect large parts, even including the internationally recognized Mt. Baekdu Biosphere Reserve (where 50–75% of the primary forests were heavily logged between 1985 and 2007; Tang et al., 2010).

Initiatives such as the transboundary protected areas and, specifically, the International Union for Conservation of Nature (IUCN) initiative “Parks for Peace,” are showing promising results in biodiversity conservation while promoting cooperation and peace-building (Vasilijević et al., 2015); the United Nations “Peace and Biodiversity Dialogue Initiative,” launched in 2015, is pursuing the same goals (https://www.cbd.int/peace/). Taking advantage of the central role of the BDDG on the heart of Koreans, we believe that an integrated strategy of conservation of natural and cultural heritage, perhaps following the spirit of the Delos initiative (http://www.med-ina.org/delos/) will offer a way forward. Ideally, the cooperative conservation efforts toward the BDDG might play a central role because these mountains are shared between South and North Korea not only physically (in a proportion of approximately 4:3), but also culturally; they are home of about half of the sacred peaks for the Koreans regardless of their religion or belief (they harbor sites holy to Shamanists, Buddhists, Daoists, Neo-confucianists, and even Christians; Mason, 2011), and are peppered with religious sites and/or objects (e.g., temples, shrines, stones, and grottoes). For example, nearly one-fifth of the Buddhist temples of South Korea are located in the BDDG (Cho and Chun, 2015). The South Korean experience in preserving its part of the range can be a valuable asset; since more than one decade ago, the BMR is effectively protecting the almost 700 km of the South Korea's section of the range, whereas the domestic legislation ensures the preservation of most of the cultural and religious sites located in the mountains. The BDDG hiking trail, 735 km long, is already a major tourism attraction in South Korea and, if managed in a sustainable way, may add impetus to an integrative conservation of the most beloved mountains on the Korean Peninsula. We hope that the recent thaw in the inter-Korean relations (after years of escalating tensions and threats of nuclear war) may pave the way to a shared strategy for preserving and restoring the BDDG.

## Author contributions

MYC, SS, GUS, CHL, JL-P, and MGC conceived the paper. JL-P and MGC wrote the paper. MYC, SH-M, CHL, JL-P, and MGC revised the paper. All authors approved it for publication.

### Conflict of interest statement

The authors declare that the research was conducted in the absence of any commercial or financial relationships that could be construed as a potential conflict of interest.
